# Supplementary material for: Advancing health equity in cancer care: The lived experiences of poverty and access to lung cancer screening
Source: PLoS One. 2021 May 6;16(5):e0251264. doi: 10.1371/journal.pone.0251264 (PMC8101716; doi:10.1371/journal.pone.0251264)
Supplement: S1 File — (PDF) [file pone.0251264.s001.pdf]

S1 File: Interview Guide - Screeners

General information

1. Tell me a little bit about yourself and your day to day?
   Probe: social identity, social determinants of health
2. How would you describe your health?
3. At what age did you start smoking?
4. Till when/ or current frequency
5. If stopped: when, and why, supports

Lung cancer screening

1. Why do you think your doctor thought lung cancer screening might be a good idea for you?
2. What influenced your decision to go for the test?
   a. Personal (physical, financial, time)
   b. Provider (trust, communication)
   c. System (accessibility, opportunity)
3. Can you tell me more about your conversation with your healthcare provider?
4. Was there anyone who influenced you in your decision to get screened?
5. Can you describe your experience of the process of lung cancer screening?
   a. Positive/ negative experiences
   b. Barriers that had to be overcome
   c. Facilitators to process
6. What are the benefits of lung cancer screening from your perspective?
7. What factors do you think will influence peoples decisions to be screened?
   a. Personal
   b. Provider
   c. System
8. What can make it easier for people to undergo screening in general?
   a. Personal
   b. Provider
   c. System (location, procedures, false positives, risks, hours)
9. Have you ever participated in any other type of cancer screening?

Closing

- Thank you, these are the questions which I had for you today. Is there anything else you would like to comment on or add before we close this interview?
