# Supplementary material for: Advancing health equity in cancer care: The lived experiences of poverty and access to lung cancer screening
Source: PLoS One. 2021 May 6;16(5):e0251264. doi: 10.1371/journal.pone.0251264 (PMC8101716; doi:10.1371/journal.pone.0251264)
Supplement: S2 File — (PDF) [file pone.0251264.s002.pdf]

S2 File: Interview Guide – Nonscreeners

General information

1. Tell me a little bit about yourself and your day to day?
   Probe: social identity, social determinants of health
2. How would you describe your health?
3. At what age did you start smoking?
4. Till when/ or current frequency
5. If stopped: when, and why, supports

Lung cancer screening

1. Why did you chose not to be screened for lung cancer at this time?
   - Personal
   - Provider level
   - Structural/ system level
2. Would you consider screening in the future if these issues were to change?
3. What are the most important barriers to going for preventative health checks such as lung cancer screening?
   - Personal
   - Provider
   - System
4. From your perspective, what can we do to make it easier for patients to seek preventative healthcare such as lung cancer screening?
   - Personal
   - Provider
   - System

Closing

- Thank you, these are the questions which I had for you today. Is there anything else you would like to comment on or add before we close this interview?
