# Supplementary material for: Advancing health equity in cancer care: The lived experiences of poverty and access to lung cancer screening
Source: PLoS One. 2021 May 6;16(5):e0251264. doi: 10.1371/journal.pone.0251264 (PMC8101716; doi:10.1371/journal.pone.0251264)
Supplement: S3 File — (PDF) [file pone.0251264.s003.pdf]

S3 File: Consolidated criteria for reporting qualitative studies (COREQ) [1]

Item	Description	Response	Reported in section and page number or not applicable N/A
**Domain 1: Research team and reflexivity**			
Personal Characteristics			
1. Interviewer/facilitator	Which author/s conducted the interview or focus group?	AS conducted all of the interviews	Methods, data collection, page 8
2. Credentials	What were the researcher's credentials? E.g. PhD, MD	AS- MD, MSc, PhD;<br>AL – MD, PhD, CCFP;<br>MV- PhD, MHsc, RN<br>MAO –PhD<br>SH – MD, MPH<br>PS- MBBS, MHSc, CCFP(AM)<br>GL – MD, MSc, FRCPC<br>MG - MBBS, FRCPC, Med<br>LE - MD<br>EN - MHSc	Methods, page 9
3. Occupation	What was their occupation at the time of the study?	AS – Lead Qualitative Researcher;<br>AL, SH, PS, GL – Clinician Scientist;<br>MAO – Qualitative	Methods, page 9

		researcher;<br><br>MV – Mixed methods researcher;<br><br>EN – Federal health care administrator	
4. Gender	Was the researcher male or female?	Female: AS, AL, MV, MAO, EN, MG;<br><br>Male: SH, GL, LE, PS	Principal author gender – page 9
5. Experience and training	What experience or training did the researcher have?	AS, MAO are experienced qualitative researchers<br><br>MV is a mixed methods researcher;<br><br>AL and GL are clinical epidemiologists;<br><br>MG and LE are clinical investigators;<br><br>SH is researcher on homelessness and health<br><br>PS is a quantitative researcher in smoking cessation<br><br>EN has experience in policy, program development and health services delivery.	Principal author and last author experience - Methods, page 9
Relationship with participants			

6. Relationship established	Was a relationship established prior to study commencement?	No prior association with participants existed prior to study commencement.	Methods, participant recruitment, page 7
7. Participant knowledge of the interviewer	What did the participants know about the researcher? e.g. personal goals, reasons for doing the research	Participants were given information about the study at the time of recruitment.	Methods, participant recruitment, page 7
8. Interviewer characteristics	What characteristics were reported about the interviewer/facilitator? e.g. Bias, assumptions, reasons and interests in the research topic	AS and AL conduct research on health inequities. This is reported in the manuscript.	Methods, data analysis, page 9
**Domain 2: study design**			
Theoretical framework			
9. Methodological orientation and Theory	What methodological orientation was stated to underpin the study? e.g. grounded theory, discourse analysis, ethnography, phenomenology, content analysis	Qualitative study with theory informed thematic analysis.	Methods, study design, page 4
Participant selection			
10. Sampling	How were participants selected? e.g. purposive, convenience, consecutive, snowball	Screeners were recruited through purposive sampling; nonscreeners were recruited through derived rapport.	Methods, participant recruitment, page 7
11. Method of approach	How were participants approached? e.g. face-to-face, telephone, mail, email	Participants were approached face-to-face.	Methods, participant recruitment,

			page 7
12. Sample size	How many participants were in the study?	18 participants were included in the study.	Results, page 10
13. Non-participation	How many people refused to participate or dropped out? Reasons?	None of the participants dropped out.	N/A
Setting			
14. Setting of data collection	Where was the data collected? e.g. home, clinic, workplace	Data was collected through telephone/ face-to-face interviews and field notes.	Methods, data collection, page 8
15. Presence of non-participants	Was anyone else present besides the participants and researchers?	Interviews were done via directly with participant.	Methods, data collection, page 8
16. Description of sample	What are the important characteristics of the sample? e.g. demographic data	Sociodemographic data of participants is reported.	Results, page 10
Data collection			
17. Interview guide	Were questions, prompts, guides provided by the authors? Was it pilot tested?	The interview guide is included in the supplementary files.	Methods, data collection, page 7 & supplementary file 1 and 2
18. Repeat interviews	Were repeat interviews carried out? If yes, how many?	Repeat interviews were not carried out.	N/A
19. Audio/visual recording	Did the research use audio or visual recording to collect the data?	Audio-recording was used.	Methods, data collection, page 8
20. Field notes	Were field notes made during and/or after the	Field notes were made by AS during the	Methods, data collection, page

	interview or focus group?	interviews	8
21. Duration	What was the duration of the interviews or focus group?	Approximately 40 – 60 minutes	Methods, data collection, page 8
22. Data saturation	Was data saturation discussed?	Yes, conceptual saturation is discussed.	Methods, data collection, page 8
23. Transcripts returned	Were transcripts returned to participants for comment and/or correction?	No. The transcripts were not returned to participants. We used peer debriefing in place of member checking.	Methods, data analysis, page 9
**Domain 3: analysis and findings**			
Data analysis			
24. Number of data coders	How many data coders coded the data?	AS and AL coded the data.	Methods, data analysis, page 9
25. Description of the coding tree	Did authors provide a description of the coding tree?	Coding tree was developed based on conceptual framework of the study	Methods, data analysis, page 9
26. Derivation of themes	Were themes identified in advance or derived from the data?	Themes were identified from the data.	Methods, data analysis, page 9
27. Software	What software, if applicable, was used to manage the data?	NVivo Version 12 was used.	Methods, data analysis, page 9
28. Participant checking	Did participants provide feedback on the findings?	The participants did not provide feedback on the findings.	N/A
Reporting			
29. Quotations presented	Were participant quotations presented to	Yes. Each quotation is identified by the	Results, pages 10-15

	illustrate the themes / findings? Was each quotation identified? e.g. participant number	pseudonym of the participant.	
30. Data and findings consistent	Was there consistency between the data presented and the findings?	Consistency between the data and findings.	Table 2
31. Clarity of major themes	Were major themes clearly presented in the findings?	The major themes are clearly presented.	Table 2
32. Clarity of minor themes	Is there a description of diverse cases or discussion of minor themes?	Minor themes are discussed including outlier themes.	Strengths and limitations, page 20

Reference: Tong A, Sainsbury P, Craig J. Consolidated criteria for reporting qualitative research (COREQ): a 32-item checklist for interviews and focus groups International Journal for Quality in Health Care 2007;19:349–357
